# The Challenge of Debunking Health Misinformation in Dynamic Social Media Conversations: Online Randomized Study of Public Masking During COVID-19

**DOI:** 10.2196/34831

**Published:** 2022-03-02

**Authors:** Mehdi Mourali, Carly Drake

**Affiliations:** 1 Haskayne School of Business University of Calgary Calgary, AB Canada; 2 Department of Management and Marketing North Central College Naperville, IL United States

**Keywords:** misinformation, debunking, correction, social media, truth objectivity, COVID-19, infodemiology, health information, digital health, public health, health professional

## Abstract

**Background:**

The spread of false and misleading health information on social media can cause individual and social harm. Research on debunking has shown that properly designed corrections can mitigate the impact of misinformation, but little is known about the impact of correction in the context of prolonged social media debates. For example, when a social media user takes to Facebook to make a false claim about a health-related practice and a health expert subsequently refutes the claim, the conversation rarely ends there. Often, the social media user proceeds by rebuking the critic and doubling down on the claim.

**Objective:**

The aim of this study was to examine the impact of such extended back and forth between false claims and debunking attempts on observers’ dispositions toward behavior that science favors. We tested competing predictions about the effect of extended exposure on people’s attitudes and intentions toward masking in public during the early days of the COVID-19 pandemic and explored several psychological processes potentially underlying this effect.

**Methods:**

A total of 500 US residents took part in an online experiment in October 2020. They reported on their attitudes and intentions toward wearing masks in public. They were then randomly assigned to one of four social media exposure conditions (misinformation only vs misinformation+correction vs misinformation+correction+rebuke vs misinformation+correction+rebuke+second correction), and reported their attitudes and intentions for a second time. They also indicated whether they would consider sharing the thread if they were to see it on social media and answered questions on potential mediators and covariates.

**Results:**

Exposure to misinformation had a negative impact on attitudes and intentions toward masking (β=–.35, 95% CI –.42 to –.29; *P<*.001). Moreover, initial debunking of a false claim generally improved attitudes and intentions toward masking (β=.35, 95% CI .16 to .54; *P<*.001). However, this improvement was washed out by further exposure to false claims and debunking attempts (β=–.53, 95% CI –.72 to –.34; *P<*.001). The latter result is partially explained by a decrease in the perceived objectivity of truth. That is, extended exposure to false claims and debunking attempts appear to weaken the belief that there is an objectively correct answer to how people ought to behave in this situation, which in turn leads to less positive reactions toward masking as the prescribed behavior.

**Conclusions:**

Health professionals and science advocates face an underappreciated challenge in attempting to debunk misinformation on social media. Although engaging in extended debates with science deniers and other purveyors of bunk appears necessary, more research is needed to address the unintended consequences of such engagement.

## Introduction

### Context

The internet in general and social media in particular have become important sources of information for many people seeking medical and health-related information. As of 2014, 72% of internet users in the United States reported having searched for health-related information online [1]. More recently, 49% of US adults reported obtaining at least some of their news about the COVID-19 vaccine on social media, and among those who regularly obtain news from social media, 61% rated social media as an important way of keeping up with news about COVID-19 vaccines [2]. Yet, content disseminated through social media sites such as Facebook, Twitter, or Reddit remains largely unregulated, and is replete with false and misleading information [3-8].

The widespread availability and consumption of false, inaccurate, or incomplete health information (herein referred to as “health misinformation”) is a serious problem that can cause individual and social harm by promoting erroneous beliefs about health and illness, leading to detrimental behavior [9,10]. For example, the persistent circulation of unfounded claims linking vaccination to autism has convinced many parents not to immunize their children, which has resulted in a significant rise in vaccine-preventable diseases and death [11]. Believing misinformation about COVID-19 has been linked to lower adoption of health protective behaviors [12] and greater consumption of harmful products [13]. Evidence also suggests that online disinformation campaigns on a global scale have played a key role in the notable drop in vaccination coverage over time [14].

Recognizing the potentially disastrous consequences of letting misinformation proliferate on social media, researchers from diverse fields have proposed a variety of countermeasures, including technological solutions aimed at limiting exposure to misinformation, educational interventions aimed at empowering people to recognize and deal with misinformation, as well as communication tools designed to help debunk and correct misinformation [15-19]. Importantly, health experts and health care professionals have been called upon to play an active role in correcting health misinformation when they encounter it on social media [20-27].

Elsewhere, a rich literature on debunking has shown that properly designed corrections can be effective at countering misinformation [28-31]. This research, however, has examined the issue mostly from a static perspective. In a typical study, participants are first exposed to misinformation. Subsequently, some participants receive a correction, and their responses are compared to a control group that received no correction or a comparison group that received an alternate correction varying in its content, source, or some other relevant attribute (eg, [27,32-36]). Although this paradigm allows for a clean test of the relative effectiveness of specific debunking interventions, it oversimplifies the dynamic nature of social media conversations. For example, when a social media user takes to Facebook to make a false claim about a health-related practice, and a health expert subsequently refutes the claim, the conversation rarely ends there. Often, the social media user proceeds by rebuking the critic and doubling down on the claim.

### Objectives

The aim of this study was to examine the impact of such extended back and forth between false claims and debunking attempts on observers’ dispositions toward behavior favored by science. The US Centers for Disease Control and Prevention (CDC) had been recommending mask wearing in public since April 2020. However, by the time of our study in October 2020, less than half of the states had issued a mandate for mask wearing in public [37], and misinformation regarding the safety and efficacy of masking had become rampant on social media [38].

In this study, we tested competing predictions about the effect of extended exposure on people’s attitudes and intentions toward masking in public and explored several psychological processes potentially underlying this effect.

On the one hand, properly debunking a false claim may have a lasting effect, such that when the purveyor of misinformation proceeds to rebuke the critic and double down on the false claim, observers’ attitudes toward masking would remain unaffected by the new round of misinformation. A detailed refutation that includes a clear explanation of why a claim is false and what is true instead [16,39] could have a persistent impact on observers’ attitudes because, in addition to arming them with facts, it undermines the credibility of the argument underlying the false claim [40]. Moreover, research on “prebunking” has shown that it is possible to inoculate individuals against misinformation before it is even encountered [41-43]. Extending the principle of prebunking to prolonged social media debates, one could surmise that witnessing a thorough refutation of a false claim early in the debate may inoculate people against later assertions of the false claim.

On the other hand, repeated exposure to misinformation and its rebuttal could create uncertainty about the very existence of true facts [4,44]. For example, in reference to President Donald Trump’s extensive record of making false and misleading claims, Lewandowski et al [44] quoted a 2017 editorial from the Bangor Daily News suggesting that one important consequence of the repeated falsehoods is that “A third of the population will say ‘clearly the White House is lying,’ a third will say ‘if Trump says it, it must be true,’ and the remaining third will say ‘gosh, I guess this is unknowable’.” A year later, former Director of National Intelligence, James Clapper [45], warned that “many Americans are questioning if facts are even knowable, as foreign adversaries and our national leaders continue to deny objective reality while advancing their own alternative facts.”

In the health domain, exposure to conflicting information about a wide range of topics, including mammography [46,47], nutrition [48,49], and the human papillomavirus vaccine [50], has been shown to increase confusion, uncertainty, and negative attitudes toward the health topic in question. Together, these results suggest that exposure to an extended back and forth between false claims and debunking attempts may generate doubt about the very existence of a nonsubjective true answer to a health-related question. This study specifically examined the discourse surrounding the question of whether wearing masks in public should be prescribed. Based on extant theory, we hypothesized that such discourse would weaken people’s attitudes and intentions toward masking in public as the prescribed behavior.

In addition to testing these competing predictions, we explored the impact of extended exposure on people’s intentions to share the social media threads. This has direct implications for understanding how misinformation spreads on social media.

## Methods

### Participants and Procedure

Respondents were recruited from Prolific, a platform for recruiting online participants that explicitly caters to researchers. Prolific provides diverse and valid samples, and its data quality compares favorably with that of other online platforms such as MTurk [51,52]. US residents with an existing account on Prolific took part in the study on October 16, 2020, in exchange for monetary compensation.

Upon consenting in writing, participants answered questions about the relevance of various sources of health information, the impact of COVID-19 (including their perceived risk of infection), and reported on their attitudes and intentions toward wearing masks in public. They then completed a first attention check and were randomly assigned to one of four social media exposure conditions (misinformation only [M] vs misinformation+correction [MC] vs misinformation+ correction+rebuke [MCR] vs misinformation+correction+ rebuke+second correction [MCRC]). The attention check consisted of a statement at the end of a question asking participants to choose a specific answer to ensure they were reading the questions.

**Figure 1:**
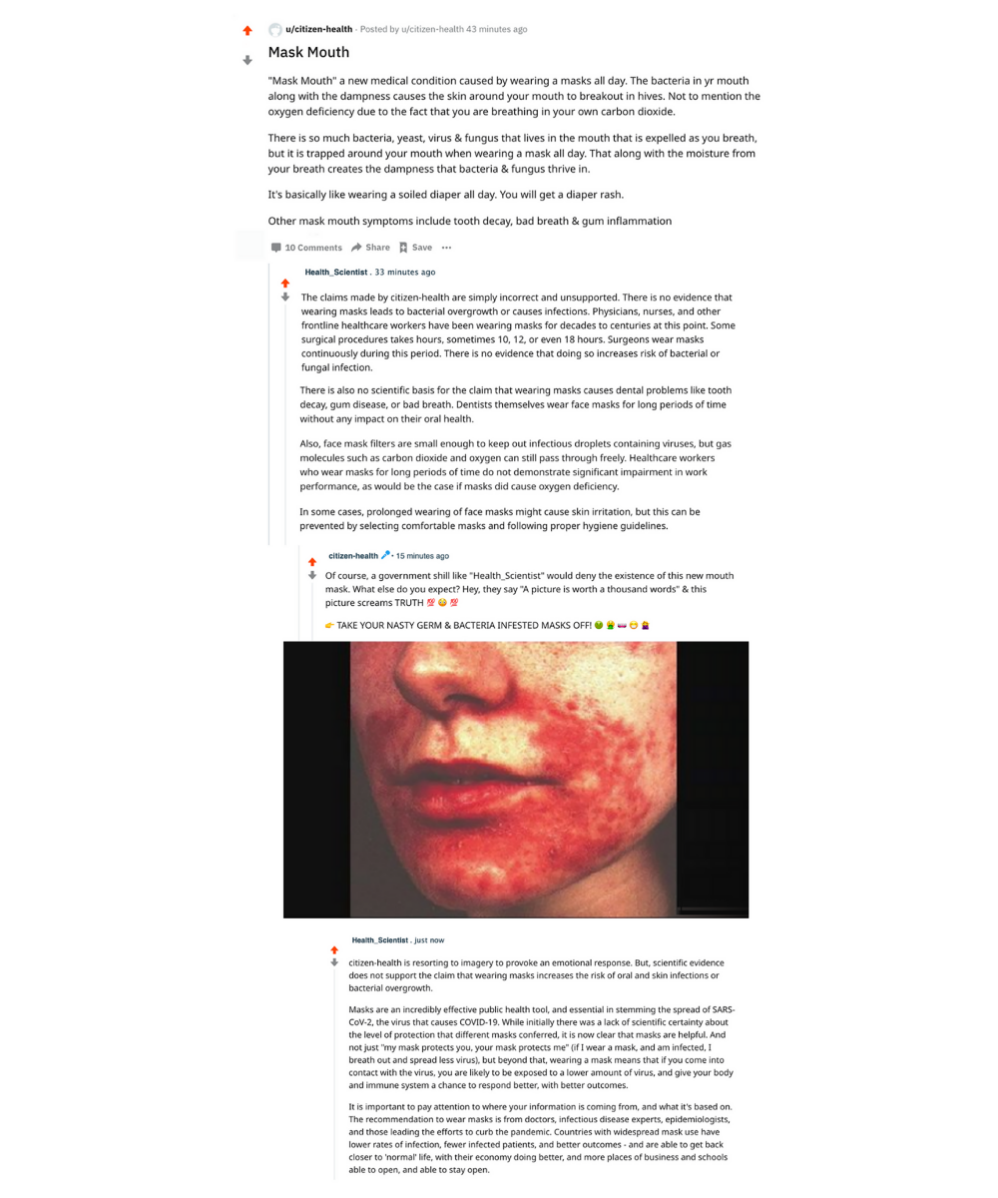
Reddit posts for the misinformation+correction+rebuke+second correction condition.

The social media content consisted of Reddit posts that were adapted from real social media posts and expert responses collected by the International Fact Checking Network [38], which are shown in full in Figure 1. Participants in the M condition reviewed a post by a user with the username *citizen-health* arguing that wearing face masks can cause a new disease called “mask mouth.” Those in the MC condition saw the same post plus a correction from a user with the username *Health_Scientist*, pointing out that there is in fact no scientific evidence of any new disease caused by wearing face masks. Those in the MCR group saw a thread containing the previous two posts plus a second post from *citizen-health* rebuking *Health_Scientist* and doubling down on the original claim. Finally, those in the MCRC condition saw a thread containing the previous three posts plus a second correction from *Health_Scientist*.

After reviewing the Reddit thread, participants completed a comprehension check assessing their comprehension of the position argued by *citizen-health*. They then indicated whether they would consider sharing the thread if they were to see it on social media, and reported their attitudes and intentions for a second time. They also answered questions on potential mediators and additional covariates. Mediators included perceptions of objectivity of truth, argument strength, and warmth and competence of the protagonists in the Reddit exchange. Covariates included perceived COVID-19 risk, cognitive reflection, conspiracy mentality, and political orientation.

### Ethical Considerations

The study was approved by the University of Calgary Conjoint Faculty Research Ethics Board (REB20-1178) and was conducted according to the principles expressed in the Declaration of Helsinki.

### Measures

Attitudes were measured using three items (“Masking in public is necessary; good; beneficial”) rated on 7-point scales (1=*strongly disagree*, 7=*strongly agree*). Participants indicated their intention to wear a mask in public using a single item (“Over the next months, how often do you intend to wear a face mask when in public?”) also on a 7-point scale (1=*never*, 7=*all the time*). Since the attitude and intention measures were highly correlated, we combined them into a single index of disposition toward masking in public (α=.96 preexposure and α=.97 postexposure).

To measure perceived objectivity of truth, we asked respondents to consider the question “Should people wear masks in public*?*” and indicate the extent to which they think there is an objectively true answer to this question [53,54]. They reported their answers on a 7-point scale (1=*definitely no objective truth*, 7=*definitely an objective truth*).

Perceived argument strength was measured using five items adapted from Zhao et al [55]. A sample item is “The arguments of citizen-health are a convincing reason against masking in public” (1=*strongly disagree*, 7=*strongly agree*). The complete scale (α=.86 for *citizen-health* and α=.94 for *Health_Scientist*) is detailed in Multimedia Appendix 1.

Participants rated the warmth and competence of both protagonists on 11-point bipolar scales adapted from previous research [40,56]. Perceived warmth was assessed using four items (α=.93 for *citizen-health* and α=.96 for *Health_Scientist*), including *unfriendly*/*friendly*, *cold*/*warm*, *irritable*/*good-natured*, *unsympathetic*/*sympathetic*. Perceived competence was assessed using six items (α=.97 for *citizen-health* and α=.98 for *Health_Scientist*), including *uninformed*/*informed*, *unqualified*/*qualified*, *unreliable*/*reliable*, *unbelievable*/*believable*, and *incompetent*/*competent*.

Respondents indicated the probability they will be infected with the coronavirus in the next 12 months on a sliding scale (0=0%, 100=100%), and rated how harmful it would be for their health if they were to become infected (1=*not at all*, 5=*extremely*). We computed a perceived COVID-19 risk score by multiplying the probability of infection by the perceived harm and dividing by 100.

Conspiracy mentality was measured using five items (α=.89) rated on 7-point scales (1=*strongly disagree*, 7=*strongly agree*) adapted from Bruder et al [57]. A sample item is “Events which superficially seem to lack a connection are often the result of secret activities.”

We assessed cognitive reflection by combining the three items of Frederick’s [58] original Cognitive Reflection Test (CRT; eg, “A bat and a ball cost $1.10 in total. The bat costs $1.00 more than the ball. How much does the ball cost?”) with the four items of Thomson and Oppenheimer’s [59] nonnumeric CRT (eg, “If you’re running a race and you pass the person in second place, what place are you in?”). Answers were coded 1 for a correct answer and 0 for an incorrect answer. The final cognitive reflection score is the sum of all the correct answers.

The following two items (*r*=0.89) adapted from Schmid and Betsch [40] were used to measure political orientation: (1) If you think about your own political views, where would you classify your views on this scale? (1=*very conservative*, 7=*very liberal*), and (2) If you think about your own political identity, where would you classify your views on this scale? (1=*Republican*, 7=*Democrat*). Scores were reversed so that higher scores indicate political conservatism.

Participants rated their sharing intention on a single item: “If you were to see this post on social media, would you consider sharing it?” (1=*definitely not*, 5=*definitely yes*).

### Statistical Analysis

#### Disposition Toward Masking in Public

Data analysis was performed using the statistical program R, version 4.0 [60], and the level of statistical significance was set at α=.05. To answer our main research question, we examined how progressive exposure to false claims and debunking attempts affects people’s attitudes and intentions toward masking in public. Specifically, we tested whether a change in disposition toward masking in public varied from one exposure condition to the next.

Given the structure in our data (each participant provided two sets of ratings), we fit a linear mixed-effects model with disposition toward masking in public as the outcome variable; random intercepts for participants (ID); and fixed effects for exposure condition (contrast-coded using repeated contrasts), time of rating (contrast-coded using treatment contrast), and their interaction. We also added perceived COVID-19 risk, cognitive reflection, political orientation, and conspiracy mentality as mean-centered covariates in the model. The model was estimated using maximum likelihood. We compared this model’s goodness of fit to a second model that was identical but did not include the condition×time interaction term. The likelihood ratio test indicated that model fit improved significantly when the interaction term was present (*χ^2^*_3_=33.71, *P<*.001), thus suggesting a significant interaction.

The *P* values for the mixed-effects model with interaction were estimated via *t* tests using the Satterthwaite approximations to degrees of freedom. Effect sizes for the fixed effects are indicated by the standardized regression coefficients (β values) and their 95% CIs

#### Perceived Objectivity of Truth

Should people wear masks in public? We speculated that during extended debates, the reiteration of false information and rebuke of experts might shake people’s confidence, not only in the veracity of any answer to the question but also in the very existence of an objectively true answer. To test this idea, we performed an analysis of covariance (ANCOVA) on perceived objectivity of truth with exposure condition as the independent variable. Our model controlled for preexposure disposition toward masking in public, perceived COVID-19 risk, cognitive reflection, political orientation, and conspiracy mentality.

#### Perceived Argument Strength

To test whether exposure to extended debates influences perceptions of the strength of *citizen-health*’s arguments, we performed an ANCOVA on perceived argument strength, with condition as the independent variable and controlling for the same set of covariates as indicated above.

#### Mediation Analysis

We examined whether multiple processes may underly the effect of exposure to misinformation and debunking attempts on the change in people’s disposition toward masking in public. Specifically, we tested the idea that correcting the original false claim may improve disposition toward masking, in part, by undermining the strength of the arguments put forth by the misinformation purveyor. Yet, further exposure may generate some doubt about the very existence of an objectively true answer, which, in turn, may weaken people’s attitudes and intentions toward masking.

We performed two parallel mediation analyses. The first focused on the difference between the MC and M conditions, whereas the second focused on the difference between the MCR and MC conditions. The mediation models included the change in disposition toward masking in public as the dependent variable, exposure condition as the independent variable, perceived objectivity as the first mediator, and perceived argument strength as the second mediator. Change in disposition toward masking in public was computed by subtracting participants’ initial disposition scores from their postexposure scores. The models were estimated using maximum likelihood with robust standard errors.

## Results

### Sample Characteristics

A total of 500 participants responded to the survey. Four participants failed the initial attention check and 17 failed the comprehension check. Of those, 6 were in the MC condition, 3 were in the MCR condition, and 8 were in the MCRC condition. After removing responses from participants who failed at least one attention check, we were left with a final sample of 479 participants. The sample’s demographic characteristics are shown in Table 1. It is worth noting that including data from participants who failed the attention or comprehension checks did not materially change the size, direction, or statistical significance of the reported effects.

**Table 1:** Sample characteristics (N=479).

Characteristic		Value
Age (years), mean (SD)		32.1 (12.3)
**Gender, n (%)**		
	Female	257 (53.7)
	Male	212 (44.6)
	Other	8 (1.7)
	Prefer not to answer	2 (0.4)
**Education, n (%)**		
	Less than high school	8 (1.7)
	High school graduate	55 (11.5)
	Some college but no degree	124 (25.9)
	Associate degree	42 (8.8)
	Bachelor’s degree	158 (33.0)
	Master’s degree	73 (15.2)
	Doctoral degree	3 (0.6)
	Professional degree	16 (3.3)
**Employment, n (%)**		
	Employed full time	183 (38.2)
	Employed part time	101 (21.1)
	Unemployed looking for work	61 (12.7)
	Unemployed not looking for work	28 (5.8)
	Retired	17 (3.5)
	Student	80 (16.7)
	Disabled	9 (1.9)

### Disposition Toward Masking in Public

**Figure 2:**
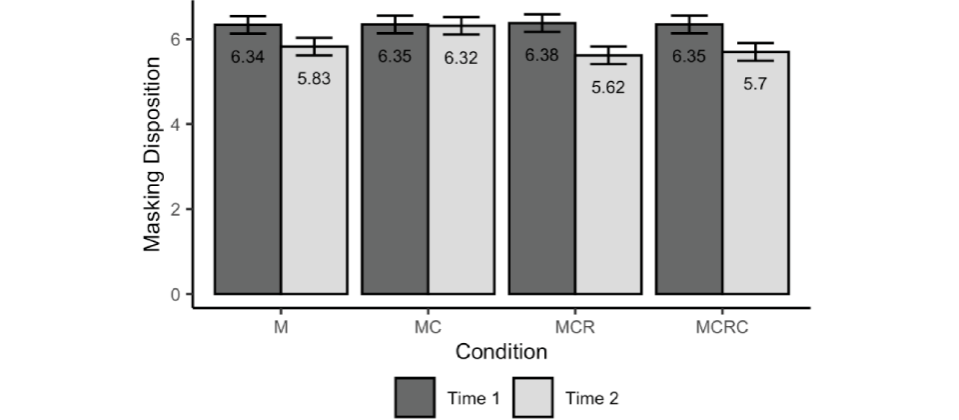
Estimated marginal means and 95% CIs for disposition toward masking in public across conditions and measurement times. M: misinformation-only experimental condition; MC: misinformation+correction experimental condition; MCR: misinformation+correction+rebuke experimental condition; MCRC: misinformation+correction+rebuke+second correction experimental condition.

Figure 2 shows the estimated marginal means and their 95% CIs. The means and SDs for all variables are presented in Table S1 of Multimedia Appendix 1. Prior to reviewing the Reddit threads, participants in all conditions reported similarly high dispositions toward masking in public. The estimated marginal means did not differ significantly between successive conditions, as shown in Table 2 (ie, *P* values for MC vs M, MCR vs MC, and MCRC vs MCR are all greater than .05). This confirmed that random assignment produced groups with equivalent baselines. The model statistics are summarized in Table 3. Furthermore, exposure to misinformation without any correction resulted in lower disposition toward masking in public (Time 2 in Table 2), while correcting false information improved disposition toward masking. Indeed, participants in the MC condition expressed more positive attitudes and intentions at time 2 than those in the M condition (Time 2: MC vs M in Table 2). Interestingly, the positive effect of correction seemed to vanish when the source of misinformation rebukes the correction and doubles down on the false claim. Participants in the MCR condition reported significantly lower disposition scores than those in the MC condition (Time 2: MCR vs MC in Table 2). Perhaps even more concerning, a second round of corrections did not appear to effectively counter the impact of the rebuke and doubling down. Indeed, disposition toward masking in public did not differ significantly between participants in the MCRC and MCR conditions.

Although not our primary focus, it is worth noting that all four covariates had significant effects in the expected directions. Disposition toward masking in public was positively related to perceived COVID-19 risk and cognitive reflection (CRT). The latter is consistent with recent research showing CRT to be negatively correlated with the perceived accuracy of fake news, and positively correlated with the ability to discern fake news from real news [61]. Conversely, disposition toward masking in public was negatively related to conspiratorial thinking and political conservatism.

**Table 2:** Fixed effects for disposition toward masking in public from the mixed-effects regression model.

Predictors	Estimate, *b* (SE)	*t* (*df*=479)	*P* value	β (95% CI)
Intercept	6.33 (0.05)	121.13	<.001	.18 (.10 to .25)
COVID-19 risk	0.15 (0.05)	3.00	.003	.11 (.04 to .18)
CRT^a^	0.06 (0.02)	2.74	.006	.10 (.03 to .17)
Political orientation	–0.31 (0.03)	–10.48	<.001	–.39 (–.46 to –.32)
Conspiracy mentality	–0.12 (0.04)	–2.99	.003	–.11 (–.18 to –.04)
Time 2	–0.49 (0.05)	–10.38	<.001	–.35 (–.42 to –.29)
MC^b^ versus M^c^	0.01 (0.15)	0.07	.94	.01 (–.20 to –.22)
MCR^d^ versus MC	0.03 (0.15)	0.22	.83	.02 (–.19 to –.24)
MCRC^e^ versus MCR	–0.03 (0.15)	–0.21	.83	–.02 (–.24 to .19)
Time 2: MC versus M	0.48 (0.13)	3.63	<.001	.35 (.16 to .54)
Time 2: MCR versus MC	–0.73 (0.13)	–5.48	<.001	–.53 (–.72 to –.34)
Time 2: MCRC versus MCR	0.11 (0.13)	0.84	.40	.08 (–.11 to .27)

^a^CRT: Cognition Reflection Test.

^b^MC: misinformation+correction.

^c^M: misinformation only.

^d^MCR: misinformation+correction+rebuke.

^e^MCRC: misinformation+correction+rebuke+second correction.

**Table 3:** Random effects of the mixed-effects regression model for disposition toward masking in public.

Random effect	Value
σ^2^	0.53
τ_00__id_	0.78
Intracorrelation coefficient	0.60
N_id_	479
Observations	958
Marginal *R*^*2*^/Conditional *R*^*2*^	0.308/0.721

### Perceived Objectivity of Truth

**Figure 3:**
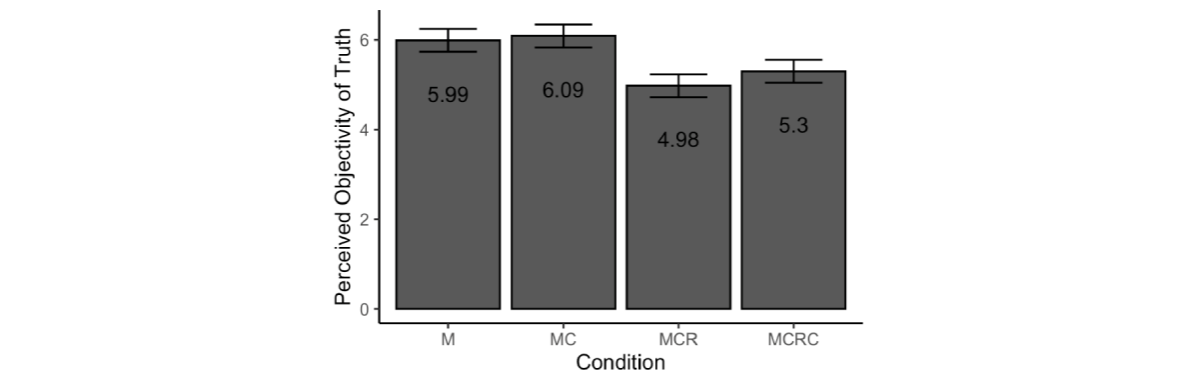
Estimated marginal means and 95% CIs for perceived objectivity of truth across conditions. M: misinformation-only experimental condition; MC: misinformation+correction experimental condition; MCR: misinformation+correction+rebuke experimental condition; MCRC: misinformation+correction+rebuke+second correction experimental condition.

The patterns in our data (see the estimated marginal means in Figure 3) lend support to the idea that reiteration of false information and rebuke of experts might weaken people’s confidence in the very existence of an objectively true answer.

We found a significant effect of condition (*F*_3,470_=16.96, *P<*.001, η_p_^2^=0.098). Planned contrasts with Bonferroni correction for multiple tests revealed that people’s perception of the objectivity of truth did not change significantly between the MC and M conditions (mean 6.18, SD 1.14 vs mean 5.91, SD 1.34, respectively; *t*_470_=0.53, *P*>.99, *d*=0.22, 95% CI –0.03 to 0.47). However, exposure to a second round of misinformation (MCR condition) resulted in appreciably lower perceptions of truth objectivity compared to those for the MC condition (mean 4.96, SD 1.88; *t*_470_=–6.01, *P<*.001, *d*=–0.79, 95% CI –1.05 to –0.52). Moreover, witnessing a second correction (MCRC condition) failed to improve perceptions of truth objectivity compared to the MCR condition (mean 5.31, SD 1.81; *t*_470_=1.74, *P=*.25, *d*=0.19, 95% CI –0.07 to 0.44). These results suggest that once undermined, perceived objectivity of truth may be difficult to restore.

With respect to the covariates, we found that initial disposition toward masking was positively related to perceived objectivity (*b*=0.31, SE 0.06; *F*_1,470_ =25.88, *P<*.001, η_p_^2^=0.05), whereas political conservatism (*b*=–0.17, SE 0.04; *F*_1,470_=14.48, *P<*.001, η_p_^2^=0.03) and conspiracy mentality (*b*=–0.12, SE 0.05; *F*_1,470_=5.06, *P=*.02, η_p_^2^=0.01) were negatively related to perceived objectivity. The effects of cognitive reflection (*b*=0.03, SE 0.03; *F*_1,470_=0.92, *P=*.34, η_p_^2^=0.002) and perceived COVID-19 risk (*b*=0.10, SE 0.07; *F*_1,470_=1.96, *P=*.16, η_p_^2^=0.004) were not significant.

### Perceived Argument Strength

The previous analysis suggested that reduction in the perceived objectivity of truth only occurs following exposure to the second round of misinformation. Thus, perceived objectivity of truth cannot account for the observed changes in attitude and intention across the entire range of exposure conditions. In particular, it cannot account for the improvement in disposition toward masking following correction of the initial false claim. Therefore, we next examined whether perceptions of the strength of *citizen-health*’s arguments may provide an alternative account.

**Figure 4:**
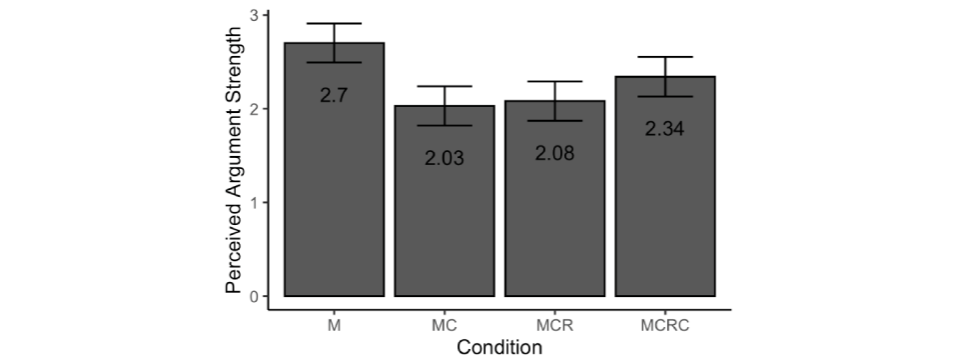
Estimated marginal means and 95% CIs for perceived argument strength across exposure conditions. M: misinformation-only experimental condition; MC: misinformation+correction experimental condition; MCR: misinformation+correction+rebuke experimental condition; MCRC: misinformation+correction+rebuke+second correction experimental condition.

Exposure condition had a significant impact on perceived argument strength (*F*_3,470_=8.21, *P<*.001, η_p_^2^=0.05). Planned contrasts with Bonferroni correction for multiple tests revealed that *citizen-health*’s arguments were perceived to be weaker in the MC condition than in the M condition (mean 1.95, SD 1.26 vs mean 2.75, SD 1.59, respectively; *t*_470_=–4.43, *P<*.001, *d*=–0.55, 95% CI –0.81 to –0.30). However, perceived argument strength remained low in the MCR condition and showed no difference from that in the MC condition (mean 2.12, SD 1.31; *t*_470_=0.34, *P*>.99, *d*=0.13, 95% CI –0.12 to 0.39). It also did not differ significantly between the MCRC condition and the MCR condition (mean 2.34, SD 1.30; *t*_470_=1.72, *P=*.26, *d*=0.17, 95% CI –0.09 to 0.42). The estimated marginal means are shown in Figure 4.

As for the covariates, political conservatism (*b*=0.14, SE 0.04; *F*_1,470_=14.03, *P<*.001, η_p_^2^=.029) and conspiracy mentality (*b*=0.16, SE 0.04; *F*_1,470_=12.19, *P<*.001, η_p_^2^=0.025) were positively related to perceived argument strength, whereas initial disposition to wearing masks (*b*=–0.39, SE 0.05; *F*_1,470_=62.96, *P<*.001, η_p_^2^=0.118) and cognitive reflection (*b*=–0.05, SE 0.03; *F*_1,470_=4.13, *P=*.04, η_p_^2^=0.009) were negatively related to perceived argument strength. Perceived COVID-19 risk (*b*=0.08, SE 0.06; *F*_1,470_=1.92, *P=*.17, η_p_^2^=0.004) was unrelated to perceived argument strength.

### Mediation Analysis

**Figure 5:**
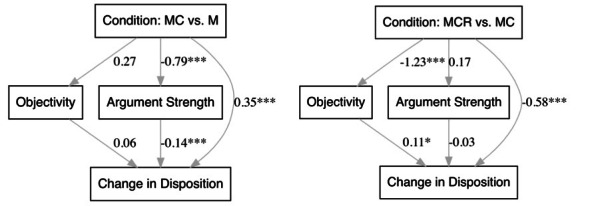
Objectivity and argument strength mediate the effect of exposure on change in disposition. Values are unstandardized coefficients (*b* values). M: misinformation-only experimental condition; MC: misinformation+correction experimental condition; MCR: misinformation+correction+rebuke experimental condition. *P<.05, ***P<.001.

When comparing the MC and M conditions, the effect of increased exposure on change in disposition toward masking in public was partially mediated by perceived argument strength (Figure 5). Indeed, both the indirect effect Condition through Argument Strength through Change in Disposition (*b*=0.12, SE 0.04, *z*=2.96; *P=*.01, 95% CI 0.04-0.19) and the direct effect Condition through Change in Disposition (*b*=0.35, SE 0.09, *z*=3.90; *P<*.001, 95% CI 0.17-0.52) were significant. However, the second indirect effect Condition through Objectivity through Change in Disposition was not significant (*b*=0.02, SE 0.02, *z*=0.93; *P=*.35, 95% CI –0.02 to 0.05). Moreover, a formal test of the difference between the indirect effects confirmed that the indirect effect through Argument Strength was significantly larger than the indirect effect through Perceived Objectivity (*b*=0.10, SE 0.04, *z*=2.84; *P=*.004, 95% CI 0.03-0.17).

Conversely, when considering the MCR and MC conditions, perceived objectivity partially mediated the effect of exposure on change in disposition. The indirect effect Condition through Objectivity through Change in Disposition was statistically significant (*b*=–0.14, SE 0.07, *z*=–2.09; *P=*.04, 95% CI –0.27 to –0.01), as was the direct effect Condition through Change in Disposition (*b*=–0.58, SE 0.13, *z*=–4.59; *P<*.001, 95% CI –0.83 to –0.33). Moreover, the indirect effect Condition through Argument Strength through Change in Disposition was not significant (*b*=–0.004, SE 0.01, *z*=–0.47; *P=*.64, 95% CI –0.02 to 0.01), and the indirect effect through Perceived Objectivity was larger than the indirect effect through Argument Strength (*b*=0.14, SE 0.07, *z*=2.00; *P=*.05, 95% CI 0.003-0.27).

These results suggest that no single process can fully account for the observed patterns in the data. Although a decrease in perceived argument strength partially explains why seeing a correction following initial exposure to misinformation improves attitudes and intentions toward masking, this path accounted for only 24.0% of the total effect of seeing a correction on change in attitudes and intentions. Moreover, a decrease in the perceived objectivity of truth partially explains why exposure to a second round of misinformation that includes rebuke of the correction and doubling down on the original false claim negatively impacts attitudes and intentions toward masking. However, this path accounted for only 19.4% of the total effect of further exposure to misinformation on change in disposition toward masking.

Given the significant direct effect of exposure on change in disposition and the modest proportion mediated in both cases, it is likely that there are additional mediators that could contribute to understanding the dynamic effects of extended exposure to misinformation on people’s attitudes and intentions toward behaviors favored by science.

### Additional Analyses

We tested for other potential mediators, but found that increased exposure did not influence participants’ perceptions of *citizen-health*’s warmth (*F*_3,470_=1.73, *P=*.16, η_p_^2^=0.01), *citizen-health*’s competence (*F*_3,470_=0.92, *P=*.43, η_p_^2^=0.006), *Health_Scientist*’s warmth (*F*_2,349_=0.63, *P=*.53, η_p_^2^=0.004), *Health_Scientist*’s competence (*F*_2,349_=0.37, *P=*.69, η_p_^2^=0.002), or strength of *Health_Scientist*’s arguments (*F*_2,349_=1.39, *P=*.25, η_p_^2^=0.008).

**Figure 6:**
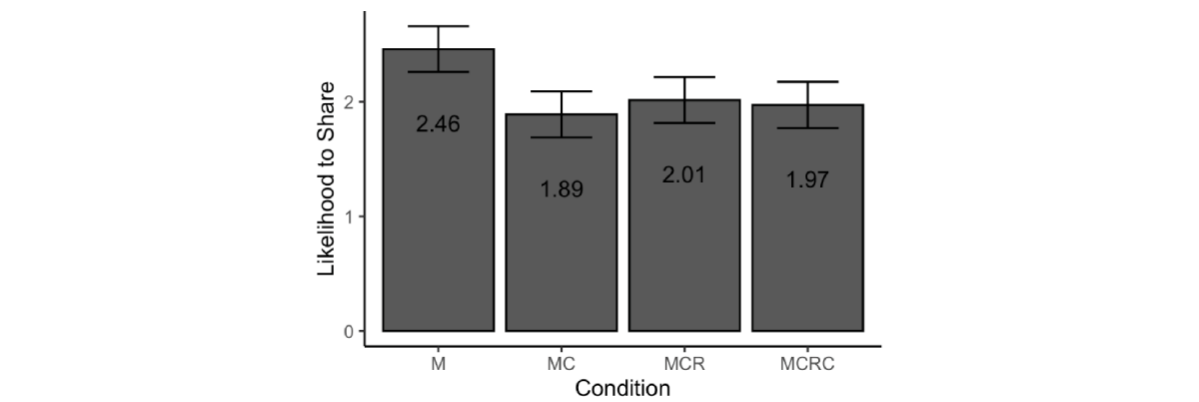
Marginal means and 95% CIs of intention to share across exposure conditions. M: misinformation-only experimental condition; MC: misinformation+correction experimental condition; MCR: misinformation+correction+rebuke experimental condition; MCRC: misinformation+correction+rebuke+second correction experimental condition.

Finally, we analyzed participants’ intention to share misinformation on social media (see Figure 6). Exposure had a significant effect on sharing intention (*F*_3,470_=6.24, *P<*.001, η_p_^2^=0.038). Planned contrasts with Bonferroni correction for multiple comparisons showed a decrease in the intention to share the original posting after seeing a correction (MC mean 1.88, SD 1.15 vs M mean 2.48, SD 1.40; *t*_470_=–3.93, *P<*.001, *d*=–0.47, 95% CI –0.72 to –0.21). Furthermore, there was no significant difference in sharing intention between the MCR and MC conditions (mean 2.03, SD 0.99; *t*_470_=0.86, *P*>.99 1, *d*=0.14, 95% CI –0.11 to 0.39), or between the MCRC and MCR conditions (mean 1.96, SD 1.09; *t*_470_=–0.29, *P*>.99, *d*=–0.07, 95% CI –0.32 to 0.19).

Examining the covariates, we found that initial disposition to wearing masks (*b*=–0.23, SE 0.05; *F*_1,470_=23.76, *P<*.001, η_p_^2^=0.046) and cognitive reflection (*b*=–0.06, SE 0.02; *F*_1,470_=6.32, *P=*.01, η_p_^2^=0.013) were negatively associated with intention to share. Neither perceived COVID-19 risk (*b*=0.06, SE 0.05; *F*_1,470_=1.33, *P=*.25, η_p_^2^=0.003) nor political orientation (*b*=0.04, SE 0.03; *F*_1,470_=1.55, *P=*.21, η_p_^2^=0.003) or conspiracy mentality (*b*=0.01, SE 0.04; *F*_1,470_ 0.03, *P=*.86, η_p_^2^=0.000) was significantly related to sharing intentions.

These results indicate that people are more likely to share misinformation when its content is consistent with their existing beliefs about the issue. Importantly, they also suggest that correcting a false claim can reduce the extent of its spread on social media, and this effect seems resistant to further exposure to the same misinformation.

## Discussion

### Principal Findings

Past research from across a variety of domains has shown that debunking misinformation works. That is, well-crafted corrections delivered by trusted sources can positively impact beliefs, attitudes, and intentions toward behavior favored by science [26-36]. With the explosion of false and misleading health claims on social media, especially since the start of the COVID-19 pandemic [62,63], scientists, experts, and health care professionals have been called upon to increase their presence on social media and help combat this “infodemic” [20-27]. A recent study found that US physicians and nurses are generally willing to take on the task even if it comes with important challenges [64]. However, little is known about the impact of extended social media debates on observers’ attitudes and intentions toward the debated issue. In this study, we tested such an impact in the context of a debate about the safety and effectiveness of wearing face masks in public during the early days of the pandemic.

We found that exposure to misinformation has a negative impact on attitudes and intentions toward masking. This result is consistent with prior research finding that exposure to misinformation negatively impacts attitudes and intentions toward behaviors favored by science [40].

Also in line with prior work [28-31], we found that initial debunking of a false claim generally improves attitudes and intentions toward masking. This effect is partially explained by a decrease in the perceived strength of the argument underlying the false claim. However, this improvement is washed out by further exposure to false claims and debunking attempts. The latter result is partially explained by a decrease in the perceived objectivity of truth. That is, extended exposure to false claims and debunking attempts appears to weaken the belief that there is a nonsubjective, correct answer to how people ought to behave in this situation, which in turn leads to less positive reactions toward masking as the prescribed behavior. Interestingly, exposure to contradictory information affects perceived truth objectivity in a nonlinear fashion. For instance, exposure to a false claim and its initial debunking does not weaken the belief that there is an objectively true answer. It appears that the level of exposure to contradictory information needs to reach a certain threshold before it affects perceived truth objectivity.

Finally, we found that people are more likely to share misinformation when its content is consistent with their existing beliefs. However, correcting misinformation reduces its likelihood of being shared on social media, and this effect persists even after multiple exposures. These results, while highlighting the value of debunking in combating the spread of misinformation on social media, suggest that, unlike attitudes, sharing intentions may be insensitive to extended exposure to a back and forth between misinformation and correction. This pattern may reflect a floor effect, in that people had expressed very low intentions to share corrected misinformation (mean of 1.89 on a 7-point scale). Exposure to further debate resulted in more negative attitudes toward masking, but did not impact sharing intentions because sharing intentions were already extremely low.

### Comparison With Prior Work

Our findings have important implications for research on debunking misinformation. Extant literature has noted that even though corrections generally reduce people’s beliefs in false information, the misinformation often continues to influence their thinking, a phenomenon known as the continued influence effect [65]. Once people process information that appears vaguely credible to them, it becomes difficult to retract it. A popular explanation of the continued influence effect assumes that people build mental models of the world and want their models to be complete. They are willing to accept false information if it allows them to build complete models. When that false information is later debunked, it creates a gap in their understanding of the world. Since people dislike gaps in their understanding and prefer their mental models to be complete, they continue to rely on information they know is false. [65,66].

Another explanation of the continued influence effect argues that attempts to correct misinformation often end up reinforcing it through repetition [67,68]. From this perspective, repeating misinformation when attempting to correct it makes it feel more familiar and fluent. By inadvertently increasing the ease with which misinformation is processed, correction attempts also increase the likelihood of it being accepted as true. Our study suggests yet another possible explanation of the stickiness of misinformation. In some situations, witnessing a heated debate with arguments for and against a controversial issue could undermine people’s confidence in the existence of an objectively true answer, which may weaken their commitment to either side of the debate.

### Limitations and Future Research

We set out to study the impact of exposure to extended debates on social media. However, our study was limited to a single platform (Reddit), and the debate was restricted to four exchanges between only two protagonists. This limits the generalizability of our findings. Interaction norms likely differ across social media platforms, which may impact how users interpret the conversation. Future research could attempt to replicate our findings using different social media platforms (eg, Facebook and Twitter), and examine the consequences of extending the debate to include more than two protagonists and more than four exchanges. Relatedly, although extended debates such as those described in this research are familiar to regular users of social media, we do not know how often they happen. Future research would benefit from quantifying the frequency of their occurrence and how it may vary across platforms.

A possible limitation of our experimental design is that the rebuke message included an image, whereas all other messages were strictly text-based. Thus, it is impossible to disentangle the impact of exposure to the rebuke message from the presence of an image. However, pinpointing which message element (image vs text) accounts for the effect of exposure was not a goal of this study. Instead, we sought to pit strong refutation against persuasive misinformation. We chose to include a graphic image in the rebuke message precisely because of its persuasive power.

Although the debunking messages used in this study were developed using recommended best practices, it is possible that different debunking techniques would have resulted in different outcomes. Future research would greatly benefit from manipulating features of the debunking argument as well as the source of debunking. For example, previous research has shown that debunking messages from anonymous social media users are more effective when they include a link to a trusted source such as the CDC [26]. Future research could test whether the positive impact of providing links to credible sources persists in the context of extended social media debates. Other features of debunking messages previously found to sometimes reduce misperceptions include the use of humor [69] and infographics [70]. However, whether such debunking techniques can be effective in the context of extended debates remains an open question.

Preemptively refuting misinformation or even just warning people that they might be misinformed has been shown to decrease later reliance on misinformation. Future research could test the effectiveness of such prebunking in the context of extended debates, where—to extend the biomedical analogy—the viral load is higher.

### Conclusions

In sum, dynamic conversations present a heretofore underappreciated challenge faced by health professionals and science advocates attempting to debunk misinformation on social media. Engaging in extended debates with science deniers and other purveyors of bunk appears necessary, but more research is needed to address the unintended consequences of such engagement.

## Appendix

Multimedia Appendix 1 Complete scales for measures and descriptive statistics.

## Abbreviations

ANCOVA: analysis of covariance
CDC: Centers for Disease Control and Prevention
CRT: Cognitive Reflection Test
M: misinformation-only experimental condition
MC: misinformation+correction experimental condition
MCR: misinformation+correction+rebuke experimental condition
MCRC: misinformation+correction+rebuke+second correction experimental condition
