# Supplementary material for: The Challenge of Debunking Health Misinformation in Dynamic Social Media Conversations: Online Randomized Study of Public Masking During COVID-19
Source: J Med Internet Res. 2022 Mar 2;24(3):e34831. doi: 10.2196/34831 (PMC8893717; doi:10.2196/34831)
Supplement: Multimedia Appendix 1 [file jmir_v24i3e34831_app1.docx]

**Multimedia Appendix 1**

## Measures

**Attitude toward masking** (1 = *strongly disagree*, 7 = *strongly agree*)

- Masking in public is necessary
- Masking in public is good
- Masking in public is beneficial

**Intention to wear a mask** (1 = *never*, 7 = *all the time*)

- Over the next months, how often do you intend to wear a face mask when in public?

**Intention to share misinformation on social media** (1 = *definitely not*, 5 = *definitely yes*)

- If you were to see this post/thread on social media, would you consider sharing it?

**Perceived objectivity of truth** (1 = *definitely no objective truth*, 7 = *definitely an objective truth*)

- Consider the question: "Should people wear masks in public?" Please tell us whether you think there is an objective true answer to this question.

**Perceived argument strength – citizen-health** (1 = *strongly disagree*, 7 = *strongly agree*)

- The arguments of citizen-health are a convincing reason against masking in public.
- The arguments of citizen-health are a believable reason against masking in public.
- The arguments of citizen-health helped me feel confident about how best to decide about masking in public.
- The arguments of citizen-health put thoughts in mind about not wanting to mask in public.
- Overall, how much do you agree or disagree with the arguments of citizen-health?

**Perceived argument strength – Health_Scientist** (1 = *strongly disagree*, 7 = *strongly agree*)

- The arguments of Health_Scientist are a convincing reason for masking in public.
- The arguments of Health_Scientist are a believable reason for masking in public.
- The arguments of Health_Scientist helped me feel confident about how best to decide about masking in public.
- The arguments of Health_Scientist put thoughts in mind about wanting to mask in public.
- Overall, how much do you agree or disagree with the arguments of Health_Scientist?

**Perceived warmth** (11-point bipolar scales)

- Unfriendly / Friendly
- Cold / Warm
- Irritable / Good-natured
- Unsympathetic / Sympathetic

**Perceived competence** (11-point bipolar scale)

- Uninformed / Informed
- Unqualified / Qualified
- Unreliable / Reliable
- Unbelievable / Believable

**Perceived COVID-19 Risk**

- How harmful would it be for your health if you were to become infected with COVID-19? (1 = *not at all*, 5 = *extremely*)
- What do you think is the probability (in percent) that you will be infected with the coronavirus in the next 12 months (sliding scale 0 = *0%*, 100 = *100%*)

**CRT**

- A bat and a ball cost $1.10 in total. The bat costs $1.00 more than the ball. How much does the ball cost? _____ cents
- If it takes 5 machines 5 minutes to make 5 widgets, how long would it take 100 machines to make 100 widgets? _____ minutes
- In a lake, there is a patch of lily pads. Every day, the patch doubles in size. If it takes 48 days for the patch to cover the entire lake, how long would it take for the patch to cover half of the lake? _____ days

**CRT2 – Non-numeric CRT**

- If you’re running a race and you pass the person in second place, what place are you in?
- A farmer had 15 sheep and all but 8 died. How many are left?
- Emily’s father has three daughters. The first two are named April and May. What is the third daughter’s name?
- How many cubic feet of dirt are there in a hole that is 3’ deep x 3’ wide x 3’ long?

**Conspiracy mentality** (1 = *strongly disagree*, 7 = *strongly agree*)

- Many very important things happen in the world, which the public is never informed about.
- Politicians usually do not tell us the true motives for their decisions.
- Government agencies closely monitor all citizens.
- Events which superficially seem to lack a connection are often the result of secret activities.
- There are secret organizations that greatly influence political decisions.

**Political orientation**

- If you think about your own political views, where would you classify your views on this scale? (1 = very conservative, 5 = very liberal)
- If you think about your own political identity, where would you classify your views on this scale? (1 = Republican, 5 = Democrat)

**Attention check**

People are very busy these days and many do not have time to follow what goes on in the government. Some do pay attention to politics but do not read questions carefully. 
To show that you have read this much, please ignore the items below about activities and instead select "Other" and type "yes" in the space next to "Other". Thank you.
   
Based on the information in the preceding paragraph, which of these activities do you engage in most regularly?

(soccer, skiing, snowboarding, running, hockey, football, swimming, tennis, cycling, other)

**Comprehension check**

What position is the Reddit user **citizen-health** arguing?

- Face masks protect against COVID-19
- Social distancing protects against COVID-19
- Face masks can cause health problems
- Social distancing can cause health problems

## Descriptive Statistics

Table S1. Observed Means and Standard Deviations for All Variables

| **Variable** | **Overall**  N = 479 | **M**  N = 122 | **MC**  N = 120 | **MCR**  N = 119 | **MCRC**  N = 118 |
| --- | --- | --- | --- | --- | --- |
| Disp_t1^1^ | 6.35 (1.25) | 6.26 (1.42) | 6.44 (0.94) | 6.35 (1.33) | 6.36 (1.28) |
| Disp_t2^2^ | 5.87 (1.45) | 5.75 (1.52) | 6.41 (1.08) | 5.59 (1.50) | 5.71 (1.52) |
| Share^3^ | 2.09 (1.19) | 2.48 (1.40) | 1.88 (1.15) | 2.03 (0.99) | 1.96 (1.09) |
| Objective^4^ | 5.59 (1.64) | 5.91 (1.34) | 6.18 (1.14) | 4.96 (1.88) | 5.31 (1.81) |
| ArgStren_CH^5^ | 2.29 (1.40) | 2.75 (1.59) | 1.95 (1.26) | 2.12 (1.31) | 2.34 (1.30) |
| ArgStren_HS^6^ | 5.63 (1.52) | - | 5.77 (1.34) | 5.42 (1.70) | 5.69 (1.48) |
| Warm_CH^7^ | 3.86 (2.27) | 4.21 (1.98) | 3.91 (2.32) | 3.61 (2.30) | 3.68 (2.45) |
| Warm_HS^8^ | 7.87 (2.32) | - | 7.83 (2.13) | 7.77 (2.55) | 8.02 (2.27) |
| Comp_CH^9^ | 2.72 (2.21) | 3.01 (2.04) | 2.41 (2.23) | 2.71 (2.34) | 2.73 (2.20) |
| Comp_HS^10^ | 8.78 (2.30) | - | 8.91 (2.14) | 8.59 (2.51) | 8.85 (2.23) |
| COVID Risk^11^ | 1.23 (1.00) | 0.97 (0.92) | 1.36 (0.97) | 1.33 (1.09) | 1.25 (0.96) |
| CRT^12^ | 3.56 (2.17) | 3.46 (2.13) | 3.43 (2.15) | 3.57 (2.24) | 3.77 (2.19) |
| CM^13^ | 4.77 (1.26) | 4.82 (1.28) | 4.54 (1.23) | 4.89 (1.30) | 4.84 (1.23) |
| PO^14^ | 2.97 (1.73) | 3.06 (1.78) | 2.78 (1.71) | 3.06 (1.70) | 2.95 (1.76) |

^1^ Disposition toward masking in public measured at time1 (before exposure to social media thread)

^2^ Disposition toward masking in public measured at time2 (after exposure to social media thread)

^3^ Intention to share misinformation on social media

^4^ Perceived objectivity of truth

^5^ Perceived argument strength for citizen-health

^6^ Perceived argument strength for Health_Scientist

^7^ Perceived warmth for citizen-health

^8^ Perceived warmth for Health_Scientist

^9^ Perceived competence for citizen-health

^10^ Perceived competence for Health_Scientist

^11^ Perceived COVID-19 risk

^12^ Cognitive reflection test

^13^ Conspiracy mentality

^14^ Political orientation
